# A Coupled Ketoreductase‐Diaphorase Assay for the Detection of Polyethylene Terephthalate‐Hydrolyzing Activity

**DOI:** 10.1002/cssc.202102750

**Published:** 2022-04-19

**Authors:** María Gimeno‐Pérez, James D. Finnigan, Coro Echeverria, Simon J. Charnock, Aurelio Hidalgo, Diana M. Mate

**Affiliations:** ^1^ Department of Molecular Biology Universidad Autónoma de Madrid Campus de Cantoblanco Madrid 28049 Spain; ^2^ Center of Molecular Biology “Severo Ochoa” (UAM-CSIC) Nicolás Cabrera 1 Madrid 28049 Spain; ^3^ Institute for Molecular Biology-IUBM Universidad Autónoma de Madrid Campus de Cantoblanco Madrid 28049 Spain; ^4^ Prozomix Ltd. Haltwhistle Northumberland NE49 9HA United Kingdom; ^5^ Institute of Polymer Science and Technology Spanish Research Council Juan de la Cierva 3 28006 Madrid Spain

**Keywords:** fluorescence, high-throughput screening, ketoreductase, plastic biodegradation, polyethylene terephthalate

## Abstract

In the last two decades, several PET‐degrading enzymes from already known microorganisms or metagenomic sources have been discovered to face the growing environmental concern of polyethylene terephthalate (PET) accumulation. However, there is a limited number of high‐throughput screening protocols for PET‐hydrolyzing activity that avoid the use of surrogate substrates. Herein, a microplate fluorescence screening assay was described. It was based on the coupled activity of ketoreductases (KREDs) and diaphorase to release resorufin in the presence of the products of PET degradation. Six KREDs were identified in a commercial panel that were able to use the PET building block, ethylene glycol, as substrate. The most efficient KRED, KRED61, was combined with the diaphorase from *Clostridium kluyveri* to monitor the PET degradation reaction catalyzed by the thermostable variant of the cutinase‐type polyesterase from *Saccharomonospora viridis* AHK190. The PET degradation products were measured both fluorimetrically and by HPLC, with excellent correlation between both methods.

## Graphical abstract

**Degrade‐detect‐validate**: A sensitive, real‐substrate, real‐time, fluorescence‐based enzymatic assay for the degradation products of polyethylene terephthalate (PET) and polyethylene furanoate (PEF) plastics was developed. It was based on the simultaneous oxidation of ethylene glycol to glyoxal by ketoreductase (KRED) and the reduction of resazurin to the highly fluorescent resorufin by diaphorase. Assay was validated against HPLC.

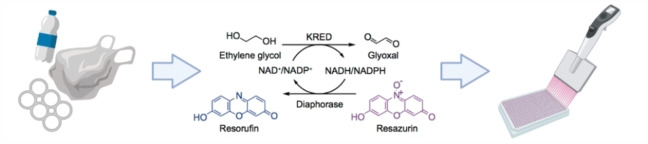

## Introduction

The term ‘plastic’ refers to an extensive family of different materials with fossil or renewable origin, although most monomers used to manufacture plastics, such as ethylene, are derived from fossil hydrocarbons.^[1]^ Plastics are present in a wide range of products used in our daily routine, e.g. food packaging, construction materials, textile fibers, electronic devices, or cosmetics, among others.[^2^, ^3^] This ubiquity is explained because plastics exhibit interesting properties such as durability, lightness, malleability, low production cost, and high thermal and chemical resistance. However, this high resistance entails a very difficult degradation, making plastic accumulation in landfills and the natural environmental one of the most serious threats to our planet.[^4^, ^5^]

Polyethylene terephthalate (PET) is the most abundant polyester plastic, with an annual production worldwide of almost 70 million tons. PET is a semi‐aromatic polyester consisting of terephthalic acid (TPA) and ethylene glycol (EG) units polymerized through ester bonds. It shows excellent physical properties such as high chemical resistance, high tensile strength, high thermal stability and transparency.^[6]^ These features make PET one of the most widely used plastics, being an essential material in the manufacture of water and soft drink bottles. However, its slow degradation time, with an estimated weight loss of 0.1–0.6 % per year in marine environments,^[7]^ causes the accumulation of huge amounts of this plastic, resulting in a serious environmental risk.

Numerous chemical degradation methods have been developed for the depolymerization of PET^[8]^ but these processes usually require harsh conditions and generate polluting by‐products. As an alternative, microorganisms or isolated enzymes could be implemented to degrade synthetic polymers.[^9^, ^10^] The biological degradation of PET is based on the hydrophilization of the fiber surface, i.e., the increase in hydrophilicity through the hydrolysis of the ends of the polymeric chains present on the surface.^[11]^ To date, less than 50 PET‐active enzymes from bacteria and fungi have been characterized.[^12^, ^13^] Relevant PET degrading enzymes include PETase and MHETase from *Ideonella sakaiensis*,^[14]^ lipase from *Candida antarctica*,^[15]^ carboxylesterase from *Thermobifida fusca*,^[16]^ *para*‐nitrobenzyl esterase from *Bacillus subtilis*,^[17]^ cutinase‐type polyesterase from *Saccharomonospora viridis* ^[18]^ or cutinases from *T. fusca*,^[19]^ *Thermobifida cellulosilytica*,^[20]^ *Humicola insolens* ^[21]^ or leaf‐branch compost cutinase.^[22]^

One of the requirements for the discovery and engineering of more efficient PET hydrolases ‐by functional metagenomics or directed evolution, respectively‐ is the availability of fast, reliable and robust screening assays that meet the “need for speed” concomitant with industrial development time frames.^[23]^ The enzymatic degradation of PET has been monitored by measuring the release of hydrolysis products such as TPA or its esters, mono(2‐hydroxyethyl)terephthalic acid (MHET) or bis(2‐hydroxyethyl)terephthalic acid (BHET). Analytical methods to quantitate the enzyme‐catalyzed degradation of PET include HPLC, titrimetric and fluorimetric assays (recently reviewed by Pirillo et al.).^[24]^ Reverse‐phase HPLC methods are based on the efficient separation of hydrolysis products on a C18 column. They allow the determination of the exact amount of each product (if suitable standards are available) but they are more time‐consuming and have a significantly lower throughput than other techniques. Titrimetric protocols are based on the addition of NaOH to counter the increasing acidity caused by the hydrolysis products of PET (TPA, MHET and BHET) in order to maintain a constant pH, thus allowing the calculation of the depolymerization yield.^[22]^ So far, only two fluorimetric assays for enzymatic polyester hydrolysis activity have been reported. First, a fluorescence‐activated droplet sorting approach for the discovery of new PET degrading enzymes has been developed that uses fluorescein dibenzoate as a fluorogenic surrogate substrate for TPA esters.^[25]^ On the other hand, a method based on the conversion of PET hydrolysis products, and thus, real substrate, to fluorescent species, such as 2‐hydroxyterephthalate from TPA by iron autoxidation. This method was validated before in 96‐well microplate using PET granulates^[26]^ and PET nanoparticles,^[27]^ but it is an endpoint assay that requires excitation at UV wavelengths, which is not a feature commonly found in ultrahigh‐throughput screening equipment, such as fluorescence‐activated cell sorting (FACS) or microfluidic on‐chip sorters.

Herein, we report a microplate fluorescence screening assay to detect PET hydrolysis products based on the coupled activity of ketoreductases (KREDs) and diaphorase. The assay is based on the oxidation of EG, MHET or BHET to the corresponding aldehydes, using NADP^+^ as cofactor. Then, the diaphorase from *Clostridium kluyveri* reoxidizes NADPH to NADP^+^, simultaneously catalyzing the reduction of resazurin to the highly fluorescent resorufin with excitation in the visible spectrum. The assay was successfully validated against HPLC using the enzymatic degradation of PET by a thermostable variant of the polyesterase Cut190 from *Saccharomonospora viridis* AHK190 as a model reaction.^[18]^

## Results and Discussion

### Assay development

**Scheme 1:**
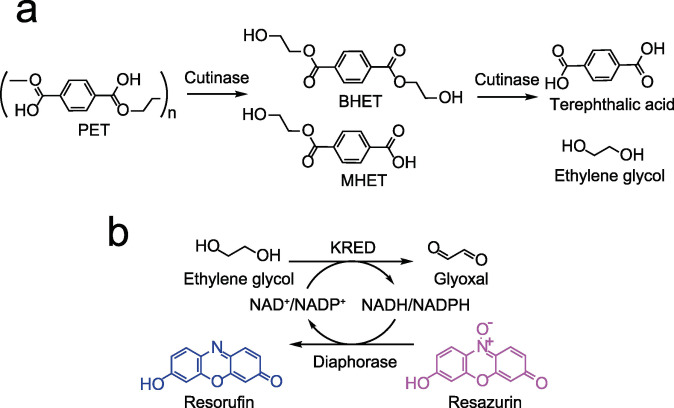
PET degradation and coupled reaction for detection of degradation compounds. a) Biodegradation of PET by cutinase releases products like BHET, MHET and their monomers, terephthalic acid (TPA) and ethylene glycol (EG). b) The coupled reaction is based on the recognition of EG by a ketoreductase (KRED) reducing the cofactor that would be recycled by a diaphorase, which will reduce non‐fluorescent resazurin to the fluorescent molecule, resorufin.

**Figure 1:**
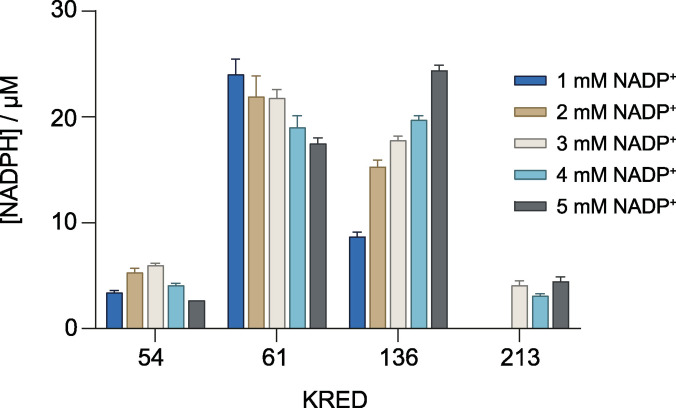
NADP^+^ conversion by KRED 54, 61, 136 and 213 using EG as substrate. NADPH formation was evaluated in triplicate at 340 nm for 60 min with different concentrations of NADP^+^ (1–5 mm) at room temperature.

**Figure 2:**
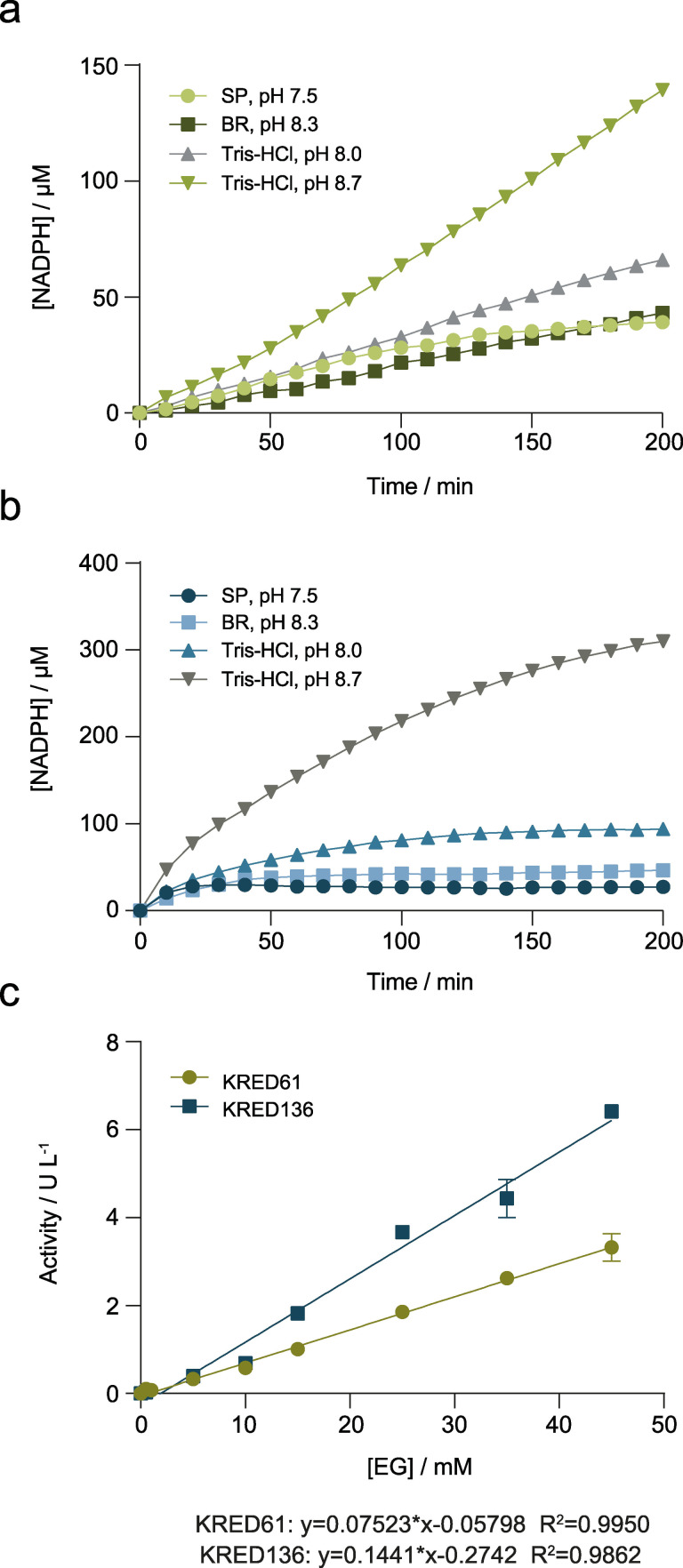
Effect of the buffer and pH in NADPH formation by KRED61 (a) and KRED136 (b). Activity of KREDs (1 mg mL^−1^ cell‐free extract) was measured in triplicate for 200 min in the presence of 3 mm of NADP^+^ at room temperature in 35 mm of the corresponding buffer. Circles: sodium phosphate buffer pH 7.5. Squares: Britton‐Robinson buffer pH 8.3. Upright triangles: Tris‐HCl buffer pH 8.0. Inverted triangles: Tris‐HCl buffer pH 8.7. c) Activity of purified KREDs at different concentrations of EG. NADP^+^ reduction was quantified in the reaction mixture with 29 and 0.5 μg of KRED61 and KRED136, respectively, and 3 mm of NADP^+^ in 35 mm Tris‐HCl buffer pH 8.0 at room temperature. SP: sodium phosphate buffer. BR: Britton‐Robinson buffer.

Ethylene glycol (EG) is a diol which, together with terephthalic acid, constitutes a basic monomer of PET plastic (Scheme 1a). The presence of hydroxyl groups makes EG a suitable substrate for ketoreductases. These enzymes oxidize EG to glyoxal in the presence of a cofactor, NAD^+^ or NADP^+^, whose recycling can be coupled with a fluorimetric or colorimetric developing reaction for added sensitivity and selectivity (Scheme 1b). The Prozomix kREDy‐to‐go panels are a set of different ketoreductases readily coupled with a colorimetric method that allows their rapid screening for activity towards diverse alcohols.^[28]^ We tested 4 kREDy‐to‐go panels ‐amounting to 384 enzymes‐ with EG as substrate, resulting in six positive hits: KREDs 54, 61, 136, 181, 213 and 296 (Figure S1). Confirmation of activity was evaluated for each positive KRED using cell‐free extracts and 45 mm EG as substrate with NAD^+^ or NADP^+^, monitoring the reduction of the cofactor at 340 nm. Only KRED61 exhibited the ability to reduce NAD^+^ producing 12.3 μm of NADH after 210 min in the presence of 5 mm oxidized cofactor (data not shown), while KREDs 54, 61, 136 and 213 showed activity with NADP^+^ (Figure 1). Under the tested conditions, KRED61 and 136 generated the highest molar concentration of NADPH, approximately 24 μm, with 1 and 5 mm NADP^+^, respectively. For further analysis, the concentration of the oxidized cofactor was fixed at 3 mm and the KREDs used were 61 and 136, which generated 22 and 17 μm of NADPH under these conditions. The nature of the buffer and pH of the reaction medium affected KRED activity with an improvement of 2‐ and 5.5‐fold in the production of NADPH at 60 min in Tris‐HCl buffer and basic pH for KRED61 and KRED136, respectively (Figures 2a and b). The time courses for the oxidation of EG with NADP^+^ with KRED61 did not reach a plateau independently of the pH and the buffer used, while the time courses with KRED136 even decreased in the case of phosphate buffer (Figures 2a and b). To exclude undesired interactions with diverse cellular compounds present in cell‐free extracts, KREDs 61 and 136 were purified to homogeneity (Figure S2b). As shown in Figure 2c, the response of both purified KREDs in the range from 5 to 45 mm EG was linear.

**Figure 3:**
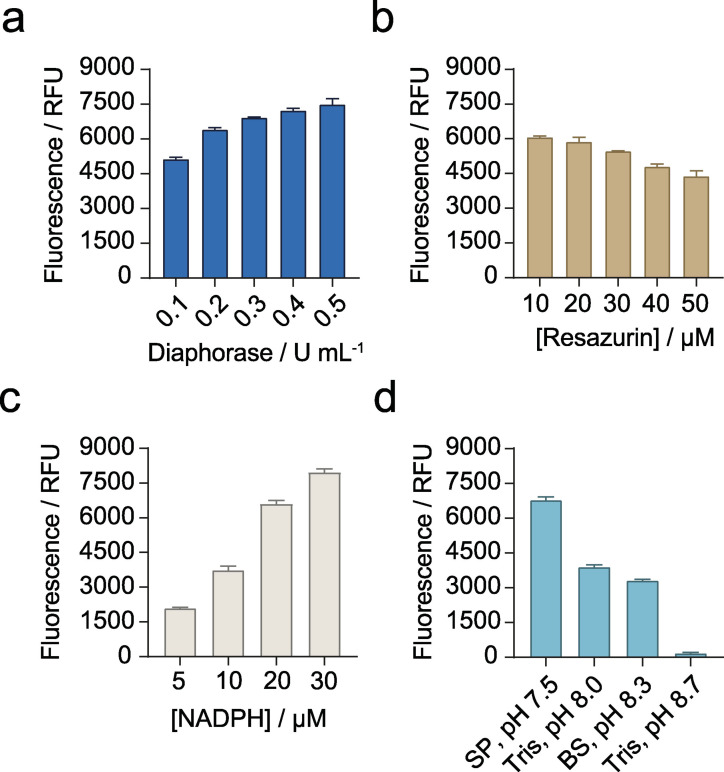
Optimization of the diaphorase reaction. Reduction of resazurin by the diaphorase with NADPH as cofactor was measured as fluorescent signal (excitation at 550 nm and emission at 580 nm) after 360 s. The concentration of diaphorase (a), resazurin (b), NADPH (c) and buffer nature (d) were evaluated. SP: sodium phosphate buffer. BR: Britton‐Robinson buffer.

To increase the sensitivity of the assay, we coupled the main KRED reaction with the conversion of a fluorogenic substrate into a highly fluorescent product, namely resazurin into resorufin. The reduction of resazurin mediated by a commercial diaphorase in presence of NADH or NADPH produces a fluorescent signal that can be measured at 580 nm after excitation at 550 nm (Scheme 1b). The reduction of both cofactors (20 μm) was tested with 0.5 U mL^−1^ of diaphorase, generating a maximum fluorescent signal of approximately 7,500 RFU for NADPH at 60 s, 1.3‐fold higher than NADH (data not shown). Therefore, the optimization of the diaphorase reaction was carried out using NADPH as cofactor and varying its concentration, the amount of enzyme, concentration of resazurin and the buffer used (Figure 3). First, the required activity of diaphorase in the reaction mix was evaluated with 20 μm NADPH and 50 μm resazurin in sodium phosphate buffer pH 7.5 showing a maximum fluorescent signal at 0.5 U mL^−1^ (Figure 3a). Considering the activity of the KRED reactions shown in Figure 2c, we considered that 0.1 U mL^−1^ of diaphorase represents at least 10‐fold excess compared to KRED activity and it would not limit the overall rate of the coupled reaction, so the next steps were optimized with this amount. The concentration of resazurin was fixed at 10 μm after testing its effect with 10–50 μm of this compound (Figure 3b). Furthermore, the resorufin calibration curve lost its linearity at concentrations higher than 30 μm (Figure S3), so the use of greater concentrations was ruled out. In contrast, the fluorescent signal incremented with increasing concentration of NADPH (Figure 3c). Finally, this set of reactions was monitored in the optimum buffer described by the manufacturer, sodium phosphate pH 7.5. As shown in Figure 3d, the highest activity was obtained in this buffer (6,777 RFU after 360 s), with almost a 2‐fold decrease in Britton‐Robinson buffer pH 8.3 and Tris‐HCl buffer pH 8.0 and virtually no activity detected in Tris‐HCl buffer pH 8.7.

**Figure 4:**
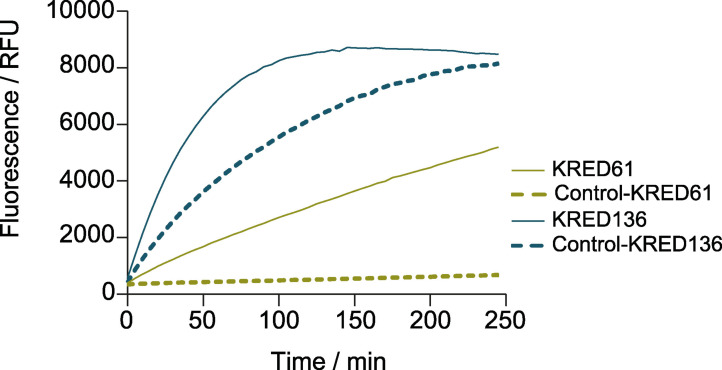
Time courses of the KRED61‐diaphorase and KRED136‐diaphorase coupled reactions with 45 mm EG as substrate. Controls without substrate were performed in parallel. Each point represents the average of three independent measurements. Standard errors were below 8 %.

To couple the KRED and diaphorase reactions, a compromise between the best conditions of the individual enzymes involved in the coupled reaction had to be reached. Thus, the selected conditions to assess the coupled reaction were: 28.7 μg KRED61 or 0.5 μg KRED136, 3 mm of NADP^+^, 0.1 U mL^−1^ diaphorase and 10 μm of resazurin in 25 mm Tris‐HCl, pH 8.0. While KRED 61 presented very low background signal in the control reaction without substrate, KRED136 showed a very high background signal, which almost equaled that of the complete reaction (Figure 4), likely due to the presence of oxidizable compounds in the commercial preparation of diaphorase. This fact led us to exclude KRED136 for further analysis and concentrate our efforts on KRED61 at the cost of increasing the assay time. The duration of the assay may yet be reduced by increasing the amount of KRED61 used or by discovery and/or engineering a more proficient KRED, which is a more permanent but also a more laborious solution. However, the linearity of the time course and the good signal: noise ratio observed in Figure 4 allowed us to reduce the assay time to 50 min and shorten the duration of subsequent stages in the optimization process with the advantage of using a readily available, commercial enzyme.

**Figure 5:**
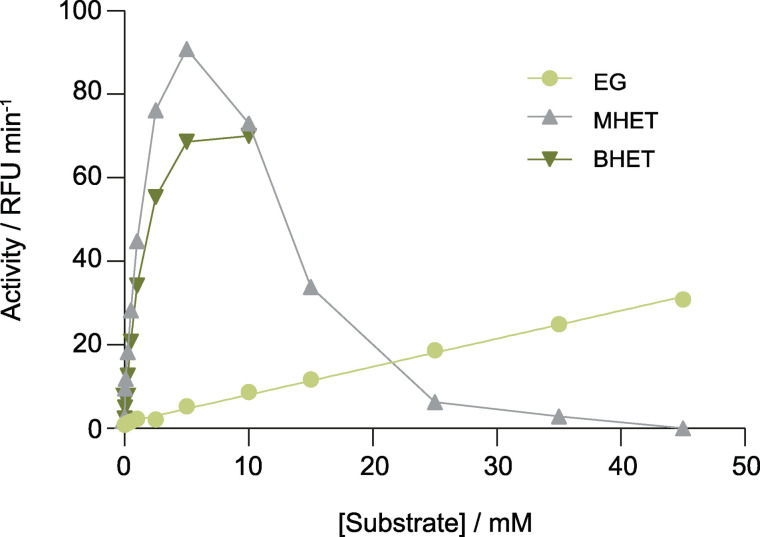
KRED61‐diaphorase coupled reaction with EG (0.05–45 mm), MHET (0.05–45 mm) or BHET (0.05–10 mm). The fluorescent emission was monitored for 50 min at room temperature in the reaction mixture composed by KRED61 (29 μg), NADP^+^ (3 mm), diaphorase (0.1 U mL^−1^) and resazurin (30 μm) in 25 mm Tris‐HCl buffer pH 8.0. Fitted line for EG is shown (y=0.6715*x+1.324, R^2^=0.9975).

The coupled reaction with KRED61 was evaluated with different concentrations of EG, increasing the resazurin concentration from 10 to 30 μm (Figure 5). Even concentrations as low as 50 μm of EG generated a fluorescent signal discernible from the control reaction, while keeping linearity at least up to 45 mm of EG with no signs of rate limitation by diaphorase. Furthermore, this represents at least a 100‐fold improvement in sensitivity compared with the direct measurement of reduced cofactors at 340 nm (Figure 2c). Although we were limited by the choice of filters available to us in our filter‐based reader, using a plate reader with monochromators, the sensitivity of the assay could likely be increased by choosing an emission wavelength closer to the 585–587 nm emission maximum of resorufin.

Other PET degradation compounds with an oxidizable hydroxyl group in their chemical structure, such as MHET and BHET, were tested as substrates in the coupled reaction (Figure 5). Both compounds were recognized by KRED61 with a 17‐ and 13‐fold higher activity at low concentrations, for example 5 mM, compared with the same concentration of EG, while at higher substrate concentrations EG was the preferred substrate. On one hand, the lack of specificity of KRED61 allows detection of any enzyme whose main products are either BHET or MHET, for example the *Is*PETase,^[29]^ the polyester hydrolase from *Pseudomonas aestusnigri* ^[30]^ and the *Fusarium* cutinase^[31]^ among others. On the other hand, the assay would not inform on the ability to hydrolyze MHET, as in for example *Is*MHETase,^[29]^ LCC metagenomic cutinase^[32]^ or TfCa from *Thermobifida fusca* KW3,^[16]^ due to the higher sensitivity of KRED61 at low concentrations towards BHET and MHET compared to EG. Unfortunately, none of the other 5 enzymes with EG‐oxidizing activity found in the initial 384 kREDy‐to‐go panel screening was selective for EG (Figure S4), which stresses the need for further metagenomic discovery or protein engineering efforts in order to obtain an EG‐selective enzyme for the specific detection of MHETase activity.

### Assay validation in the cutinase‐catalyzed degradation of PET

To validate the developed coupled assay in the degradation of PET, we monitored the hydrolysis of PET films by a thermostable variant of the polyesterase Cut190 from the thermophile *Saccharomonospora viridis* AHK190 as a model reaction using both the developed enzymatic assay and analysis by HPLC.

**Figure 6:**
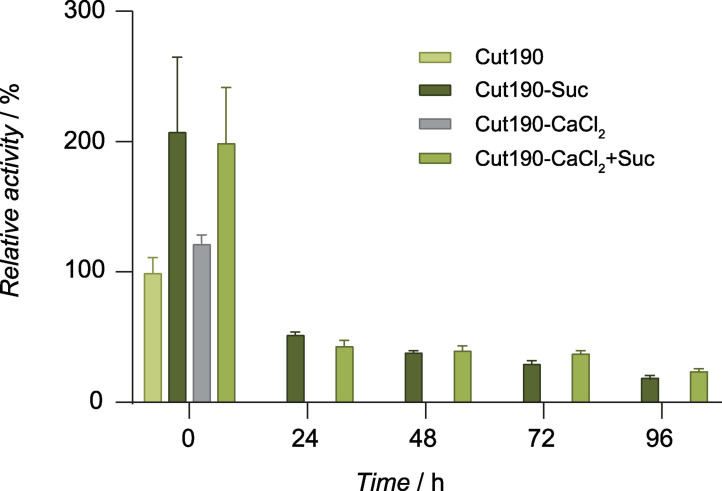
Effect of sucrose and CaCl_2_ on the thermostability of Cut190 S184P/R186S at 60 °C. Measurements were carried out in triplicate with 5 mm pNPA as a substrate in the presence and absence of 1 m sucrose and 2.5 mm CaCl_2_. Suc: Sucrose.

Cut190 S184P/R186S was reported to show PET hydrolysis activity above 60 °C.^[18]^ Although Cut190 S184P/R186S had optimal activity in the presence of 20 % (v/v) glycerol as a protein stabilizer, KRED61 was able to oxidize glycerol, which interfered with the assay (Figure S5). For this reason, we decided to test other putative stabilizers that would not interfere with the coupled assay at their final concentration. The combination of Ca^2+^ and sucrose played an important stabilizing role in Cut190 S184P/R186S, helping the enzyme keep 25 % of residual activity after 96 h at 60 °C as observed in Figure 6, without interfering with the coupled assay (Figure S5).

**Figure 7:**
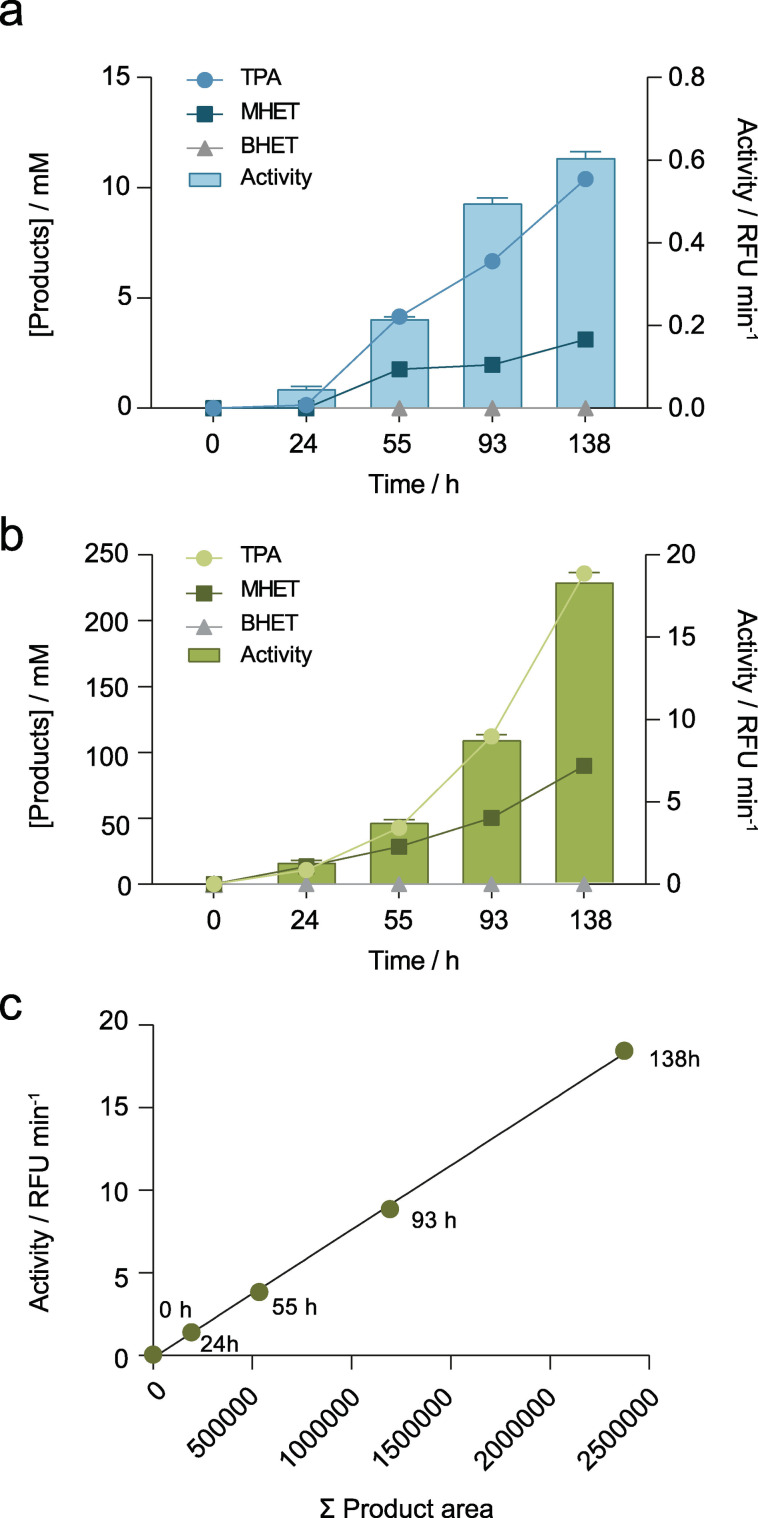
HPLC and KRED61‐diaphorase coupled reaction analysis of PET degradation products. Concentration of TPA, MHET and BHET released by degradation of PET films with Cut190 S184P/R186S at 60 °C in the absence (a) and presence (b) of 1 m sucrose is shown in the left axis measured by HPLC and the activity of the coupled reaction is indicated with bars in the right axis. c) Correlation between the sum of peak areas of TPA, MHET and BHET obtained by HPLC and the slope of the coupled KRED61‐diaphorase assay.

The enzymatic assay to detect products of PET hydrolysis was validated in a real PET degradation assay against conventional HPLC analysis using a reverse phase column with UV detection. First, calibration curves were established by HPLC for TPA, MHET and BHET from 1 to 80 μm with r^2^ values higher than 0.9997 (Figure S6). Then, the degradation of PET films by Cut190 S184P/R186S with and without sucrose at 60 °C was followed during 138 h (Figures 7a, b and S7). HPLC analysis revealed the appearance of TPA and MHET after only 24 h both in the absence and presence of sucrose. However, the release of BHET was only detected in the presence of sucrose after 138 h. In all cases, hydrolysis was favored in the presence of sucrose, with a significantly higher yield of degradation products. To confirm visually that degradation of the PET films had taken place and was the cause for the presence of the detected hydrolysis products, erosion of the film surface was evidenced by SEM after 138 h of treatment with cutinase (Figure S8). The surface was significantly more eroded when sucrose was present in the reaction, which correlates well with the higher degree of hydrolysis observed under these conditions.

As shown in Figure 7c, there was a linear relationship between the sum of peak areas corresponding to the PET hydrolysis products (TPA, MHET and BHET) and the PET hydrolyzing activity determined with the coupled KRED‐diaphorase assay, which confirms the validity of the proposed assay to detect the presence of the products of enzymatic PET hydrolysis with the different sensitivities aforementioned. Moreover, timewise, the multi‐well plate assay could allow us to set up and measure 384 assays in 2 h, which compares very favorably to the 132 h required for preparation and HPLC analysis of the same number of samples. Finally, although the assay involved many components, the reduction in assay volume concomitant to the multi‐well format also encompasses a reduction in the cost per assay. Considering the price of the reagents used (NADP^+^, KRED61, diaphorase, resazurin and Tris buffer), we calculated that the economic cost of each reaction in 96‐well plate is 7 ⋅ 10^−4^ € and 9 ⋅ 10^−5^ € in a 384‐well plate, which again compares favorably to the cost of HPLC analysis with 0.075 € per sample.

## Conclusion

The availability of high‐throughput screening methods for fast, easy and accurate determination of PET‐degrading activity is an essential requirement in metagenomic and directed evolution explorations of the natural and artificial genetic diversity, to find new or improved enzymes, respectively. The assay we presented here, based on the coupled reaction of KRED61 and diaphorase using NADP^+^ as cofactor, could detect three PET degradation products: EG, MHET and BHET in the low micromolar range, albeit with preference for BHET and MHET, and correlated well with the determination of the PET hydrolysis products by HPLC. Future work to improve this assay should focus on improving the selectivity and sensitivity towards EG to extend its applicability even further, for example to the detection of MHETases.

Most importantly, the concept proposed herein where EG and aromatic hydroxyl‐containing degradation products were detected via a KRED‐catalyzed oxidation can be extended, with the appropriate KRED, to the detection of hydrolysis of other EG‐containing polymers such as polyethylene furanoate (PEF), an emerging plastic postulated to become the replacement of PET, which can be sourced from renewable raw materials.^[33]^ Additionally, although fluorescence was chosen as a readout for sensitivity reasons, the assay could be coupled to the production of colored iodonitrotetrazolium (INT)‐formazan, in a similar format as the kREDy‐to‐go plates for easy screening of candidate PET‐hydrolyzing enzymes.

Moreover, the assay described here could be modified for implementation in ultrahigh‐throughput format based on droplet microfluidics, with the advantage of detecting real PET hydrolysis products and avoiding the use of surrogate substrates.^[25]^ To that end, the putative workflow comprised of the encapsulation of a PET nanoparticle with the library clone expressing the candidate hydrolase in water‐in‐oil (w/o) droplets prior to total[^34^, ^35^] or partial^[36]^ cell lysis. After sufficient incubation for PET hydrolysis, the reaction mix proposed in this work was added to the droplets by picoinjection[^37^, ^38^] or droplet fusion,^[39]^ following a short incubation to reveal the presence of hydrolysis products before proceeding to on‐chip droplet sorting and recovery of the DNA from the droplets by PCR (Figure S9). Although resorufin was not the optimal fluorophore for ultrahigh‐throughput screening due to its low retention in w/o droplets compared to the less “leaky” fluorescein^[40]^ or pyranine molecules,^[41]^ workflows in which resorufin was the fluorescent product of an enzymatic cascade with incubation times compatible with the proposed assay herein were described.[^42^, ^43^] Furthermore, modified resazurin substrates with improved retention in droplets are available[^44^, ^45^] and their use would be particularly suitable for this application. Alternatively, if resorufin should be avoided altogether, thanks to the flexibility or the proposed concept, a soluble formazan could be used, combined with on‐chip absorbance sorting as reported previously for dehydrogenases,^[46]^ albeit at the cost of sensitivity and throughput.^[47]^

Finally, the proposed coupled assay not only represents a concept that can be tailored to different readouts but most importantly, it can bring considerable time and cost savings for the screening of large libraries for the discovery of BHET/MHET‐releasing enzymes by functional metagenomics or directed evolution, compared with conventional HPLC analysis.

## Experimental Section

### Enzymes and reagents

Genes coding for the cutinase Cut190 S184P/R186S (S226P/R228S including the signal peptide, GenBank BAO42836.1), kREDy‐to‐go^TM^ plates and KREDs 54, 61, 136, 181, 213 and 296 were kindly provided by Prozomix Ltd. *Escherichia coli* BL21(DE3) competent cells were purchased from Stratagene.

Benzoic acid, bis(2‐hydroxylethyl) terephthalate (BHET), diaphorase from *Clostridium kluyveri*, formic acid, iodonitrotetrazolium chloride (INT), *p*nitrophenyl acetate (*p*NPA), resazurin and resorufin were purchased from Sigma (San Louis, USA). β‐Nicotinamide adenine dinucleotide (NAD^+^), β‐nicotinamide adenine dinucleotide phosphate (NADP^+^), β‐nicotinamide adenine dinucleotide reduced form (NADH) and β‐nicotinamide adenine dinucleotide phosphate reduced form (NADPH) were from Carbosynth Ltd. (Compton, UK). Benzonase nuclease was from Novagen (Madison, USA). Ethylene glycol (EG) was from May & Baker Ltd. (Dagenham, United Kingdom). Mono(2‐hydroxyethyl) terephthalate (MHET) was from Activate Scientific GmbH (Prien am Chiemsee, Germany). Sucrose was from Merck Millipore Ltd. PET films (250 μm thickness, transparent, amorphous) were purchased from GoodFellow Inc. (Wrexham, UK).

### Screening for KRED activity with EG

kREDy‐to‐go plates (Prozomix) containing 384 different KREDs were screened for activity against EG. To that end, 80 μL of a water solution containing 0.5 % EG (v/v) and 0.25 mg mL^−1^ INT were added to each well. A blank plate was also prepared by adding 80 μL of a water 0.25 mg mL^−1^ INT solution. The plates were incubated at room temperature in the dark for 24 h and absorbance at 485 nm and room temperature was recorded in a FLUOstar OPTIMA microplate reader. Positive hits were considered those with 0.2 absorbance units over the average of the blank.

### Cloning, expression and purification of KREDs

KREDs 61 and 136 were cloned in pET28a between *Nde*I and *Nhe*I. A preculture was prepared with one colony grown in 5 mL LB containing 30 μg mL^−1^ kanamycin at 37 °C and 180 rpm for 16 h. A fresh culture with OD_600_ 0.05 was prepared in 100 mL LB containing 30 μg mL^−1^ kanamycin in 500 mL flasks and grown at 37 °C and 180 rpm until the OD_600_ was 0.5. Then, IPTG was added to a final concentration of 0.5 mM, and the culture was incubated at 37 °C and 180 rpm for 7 h. Cell pellets were collected by centrifugation at 4,000 xg and 4 °C for 15 min washed with PBS and conserved at −20 °C until use. Purification of both KREDs was done as described above for cutinase Cut190 S184P/R186S and dialyzed against 20 mm Tris‐HCl buffer pH 8.0.

### Optimization of KRED reaction conditions

To test the effect of the concentration of the cofactor, the pH, and the concentration of EG on KRED activity, the generation of NADH or NADPH was measured at 340 nm and room temperature in a FLUOstar OPTIMA microplate reader.

The effect of the cofactor concentration on KRED activity was evaluated for the six positives in the kREDy‐to‐go assay with EG: KREDs 54, 61, 136, 181, 213 and 296. The assay was carried out using 45 mm EG as substrate, 0, 1, 2, 3, 4, 5 mm NAD^+^ or NADP^+^, 1 mg mL^−1^ KRED cell free extract in 35 mm sodium phosphate buffer pH 7.5.

The effect of pH on KRED activity was evaluated for KREDs 54, 61 and 136. The assay was carried out using 45 mm EG, 3 mm NADP^+^ and 1 mg mL^−1^ cell free extract of KREDs in sodium phosphate buffer pH 7.5, Tris‐HCl buffer pH 8.0, Britton‐Robinson buffer pH 8.3 or Tris‐HCl buffer pH 8.7, all 35 mm.

The effect of the concentration of EG were tested with KREDs 61 and 136. The assay was performed using 1, 2.5, 5, 10, 15, 25, 35 and 45 mm EG, 3 mm NADP^+^ and 20 μL purified enzyme with a dilution to produce a linear response, in 35 mm Tris‐HCl buffer pH 8.0.

After purification of KRED61, activity was measured using 45 mm EG, 3 mm NADP^+^, 20 μL purified enzyme with a dilution to produce a linear response in 35 mm Tris‐HCl buffer pH 8.

In all cases, appropriate negative controls without enzyme and without substrate were set up.

### Optimization of diaphorase reaction conditions

The effect of the nature of the cofactor (NADH or NADPH) on the diaphorase activity was evaluated using 0.1 and 0.5 U mL^−1^ diaphorase, 20 μm NADH or NADPH, 50 μm resazurin in 35 mm sodium phosphate buffer pH 7.5.

The effect of resazurin concentration on the diaphorase activity was using 0.1 U mL^−1^ diaphorase, 20 μm NADPH, 10, 20, 30, 40 and 50 μm resazurin in 35 mm sodium phosphate buffer pH 7.5.

The effect of NADPH concentration on the diaphorase activity was done using 0.1 U mL^−1^ diaphorase, 5, 20 and 30 μm NADPH, 10 μm resazurin in 35 mm sodium phosphate buffer pH 7.5.

The effect of the pH on the diaphorase activity was evaluated using 0.1 U mL^−1^ diaphorase, 20 μm NADPH, 10 μm resazurin in sodium phosphate buffer pH 7.5, Tris‐HCl buffer pH 8.0, Britton‐Robinson buffer pH 8.3 and Tris‐HCl buffer pH 8.7, all 35 mm.

The effect of the units of diaphorase was evaluated using 0.1, 0.2, 0.3, 0.4 or 0.5 U mL^−1^ diaphorase, 20 μm NADPH, 50 μm resazurin in 35 mm sodium phosphate buffer pH 7.5.

In all cases, the reduction of resazurin to resorufin was measured at excitation and emission wavelengths of 550 nm and 580 nm, respectively, at room temperature with a gain of 1,000 in a FLUOstar OPTIMA microplate reader.

### Coupled reaction KRED‐diaphorase

The KRED61 and diaphorase coupled reaction was assessed using commercial EG, MHET or BHET (0.05–45 mm) as substrates measuring the final formation of resorufin as described above. The reaction mixtures contained KRED 61 (29 μg), NADP^+^ (3 mm), diaphorase (0.1 U mL^−1^) and resazurin (30 μm) in 25 mm Tris‐HCl buffer pH 8.0. The effect the glycerol (0.1 % v/v) and sucrose (1 m) in the coupled reaction was evaluated under the same conditions described above and 45 mm EG.

The progress of a model PET depolymerization reaction by Cut190 S184P/R186S, in the presence and absence of sucrose, was evaluated by the coupled reaction using 20 μL of the reaction mixture from digested PET films. As aforementioned, the reduction of resazurin to resorufin was measured at room temperature with a gain of 1,000 in a filter‐based FLUOstar OPTIMA microplate reader using the 550–10 excitation and 580–10 emission filters, respectively.

### Cloning, expression and purification of cutinase

Cut190 S184P/R186S was cloned without the signal peptide and with a six‐histidine tag (His_6_ tag) at the C‐terminus in plasmid pET28a between the *Nco*I and *Xho*I restriction sites. *E. coli* BL21(DE3) was transformed with the generated pET28a‐Cut190 S184P/R186S construct. One colony was grown in Luria‐Bertani (LB) lysogeny broth (10 g L^−1^ tryptone, 5 g L^−1^ yeast extract, 5 g L^−1^ NaCl) containing 30 μg mL^−1^ kanamycin for 16 h at 37 °C and 180 rpm. Later, the culture was diluted to OD_600_ 0.05 in 100 mL lactose autoinduction medium^[48]^ with kanamycin 30 μg mL^−1^ in 500 mL flasks. Cultures were incubated at 20 °C and 180 rpm for 21 h. Absorbance was measured at 600 nm in a FLUOstar Optima microplate reader (BMG Labtech GmbH, Ortenberg, Germany). Cell pellets were collected by centrifugation at 4,000 xg and 4 °C for 15 min (5804R centrifuge, Eppendorf), washed with phosphate buffered saline (PBS) and conserved at −20 °C until use.

Subcellular localization (soluble or insoluble fraction) of the induced proteins was checked. To that end, a sample of the induced cultures were centrifuged at 4,000 xg for 15 min, resuspended in 300 μL PBS and disrupted by sonication on ice for 30 s with 0.6 s pulse and 60 % amplitude (LABSONIC M homogenizer, Sartorius). Disrupted samples were centrifuged for 10 min at 6,000 xg and supernatant (soluble) and pellet (insoluble) fractions were treated separately. Soluble fraction was centrifuged once again for 10 min at 10,000 xg, while the insoluble fraction was washed twice with Triton X‐100 0.1 %, centrifuged for 10 min at 10,000 xg and resuspended in 300 μL PBS. Both fractions were boiled at 95 °C for 10 min in Laemmli lysis buffer and analysed by SDS‐PAGE in 12 % acrylamide gels.

Cutinase Cut190 S184P/R186S was purified by affinity chromatography using a nickel‐loaded affinity resin (Ni‐NTA Superflow, Qiagen). Cell pellets (∼1.5 g) were resuspended in 30 mL lysis buffer (50 mm NaH_2_PO_4_, 300 mm NaCl and 10 mm imidazole, adjusted to pH 8.0), and 2 μL benzonase nuclease was added. Cell disruption was carried out with a pressure homogenizer (GEA Niro homogenizer) and the lysate was centrifuged at 14,000 xg and 4 °C for 30 min (Avanti J‐25 centrifuge, Beckman Coulter). Approximately 1 mL of the resin was packed, equilibrated with 10 mL lysis buffer and incubated with the cell extract in a rotating shaker for 1 h at 4 °C. Then, cell extract with the resin was packed in a plastic column and washed with 10 mL washing buffer (50 mm NaH_2_PO_4_, 300 mm NaCl and 20 mm imidazole, adjusted to pH 8.0). Finally, the bound proteins were eluted with 5 mL elution buffer (50 mm NaH_2_PO_4_, 300 mm NaCl and 300 mm imidazole, adjusted to pH 8.0).

The purified enzyme was dialyzed against 50 mm sodium phosphate buffer pH 7.5 and concentrated using Amicon Ultra‐15 centrifugal filter units with 3 kDa cut‐off (Merck Millipore Ltd., Darmstadt, Germany) in a refrigerated centrifuge.

Protein concentration was determined using a NanoDrop 1000 spectrophotometer. Protein homogeneity was analyzed by SDS‐PAGE in 12 % acrylamide gels.

### Determination of cutinase activity with pNPA

The enzymatic activity was determined at room temperature in 96‐well plate using 20 μL suitable enzyme dilutions, 210 μL 50 mm Tris‐HCl buffer pH 7.5, and 20 μL 62.5 mm *p*NPA in 8 % (v/v) DMSO. The release of *p*nitrophenolate (*p*NP) was recorded over time at 410 nm (ϵ_pNP_=13.3 mM^−1^ cm^−1^ at pH 7.5) and room temperature in a FLUOstar OPTIMA microplate reader.

### Digestion of PET films

Prior to treatment with cutinase, PET films (6 mm diameter) with a thickness of 250 μm were washed three times with Triton X‐100 5 g L^−1^, 100 mm Na_2_CO_3_ and Milli‐Q water for 30 min at 30 °C each step and dried for 18 h at 37 °C. Then, PET films were placed in a glass bottle containing 4 mL reaction volume. The reaction contained 10 μm cutinase Cut190 S184P/R186S, 2.5 mm CaCl_2_, 1 m sucrose and 100 mm Tris‐HCl buffer pH 8.0. Reactions were followed during 138 h at 60 °C and 180 rpm in a shaker, taking aliquots at 24, 55, 93 and 138 h. Reactions and control samples (without enzyme) were carried out in triplicate.

### HPLC analysis

The degradation products of PET were analyzed by HPLC with a 1525 binary pump (Waters) coupled to a Kinetex EVO C18 column (particle size 5 μm, pore size 100 Å, 150×4.6 mm) and precolumn C18, both from Phenomenex. The column temperature was kept constant at 30 °C and the injection volume was 10 μL. An acetonitrile (buffer A) and water containing 0.1 % w/v formic acid (buffer B) mixture was used as mobile phase at 0.8 mL min^−1^ during 20 min. The composition of mobile phase gradually changed from 5 % to 44 % of buffer A for 12 min, then 70 % of buffer A was maintained for 3 min followed to the initial composition until the end of analysis. Aromatic compounds were detected using a Waters 2489 UV/visible detector at 254 nm. Data were analyzed using the Breeze 2 HPLC System (Waters). Compounds were quantified on the base of peak areas using calibration curves of commercial TPA, MHET and BHET.

Samples were diluted in cold methanol and centrifuged at 10,000 xg at 4 °C for 15 min. Supernatant was filtered through a 0.45 μm nylon syringe filter (Scharlab, Barcelona, Spain).

### Scanning electron microscopy (SEM)

Polymer circles sized 6 mm in diameter were examined by SEM before and after treatment with cutinases. Cutinase‐treated samples were rinsed with 1 % SDS, with distilled water and with ethanol. PET samples were coated with gold in an argon field using a Polaron SC7640 sputter coater. SEM imaging was performed using an FEI XL30 instrument under low vacuum operating with a gaseous solid‐state detector. Imaging was performed with a beam‐accelerating voltage of 15 kV.

## Conflict of interest

The authors declare no conflict of interest.

1

## Supporting information

As a service to our authors and readers, this journal provides supporting information supplied by the authors. Such materials are peer reviewed and may be re‐organized for online delivery, but are not copy‐edited or typeset. Technical support issues arising from supporting information (other than missing files) should be addressed to the authors.

Supporting Information

## Data Availability

The data that support the findings of this study are available from the corresponding author upon reasonable request.
