# Supplementary material for: A Coupled Ketoreductase‐Diaphorase Assay for the Detection of Polyethylene Terephthalate‐Hydrolyzing Activity
Source: ChemSusChem. 2022 Apr 19;15(9):e202102750. doi: 10.1002/cssc.202102750 (PMC9321771; doi:10.1002/cssc.202102750)
Supplement: Supplementary file 1 — Supporting Information [file CSSC-15-0-s001.pdf]

ChemSusChem

Supporting Information

A Coupled Ketoreductase-Diaphorase Assay for the Detection of Polyethylene Terephthalate-Hydrolyzing Activity

María Gimeno-Pérez, James D. Finnigan, Coro Echeverria, Simon J. Charnock, Aurelio Hidalgo,* and Diana M. Mate*This publication is part of a collection of invited contributions focusing on "Biocatalysis as Key to Sustainable Industrial Chemistry". Please visit to view all contributions.© 2022 The Authors. ChemSusChem published by Wiley-VCH GmbH. This is an open access article under the terms of the Creative Commons Attribution License, which permits use, distribution and reproduction in any medium, provided the original work is properly cited.

Author Contributions

M.G.-P. Formal analysis:Lead; Investigation:Lead; Writing – original draft:Lead; Writing – review & editing:Lead
J.F. Investigation:Supporting; Resources:Supporting; Writing – review & editing:Supporting
C.E. Investigation:Supporting; Methodology:Supporting; Writing – review & editing:Supporting
S.C. Resources:Supporting; Writing – review & editing:Supporting
A.H. Funding acquisition:Supporting; Methodology:Lead; Resources:Supporting; Supervision:Supporting; Writing – review & editing:Equal
D.M. Conceptualization:Supporting; Funding acquisition:Lead; Methodology:Supporting; Project administration:Lead; Resources:Lead; Supervision:Lead; Writing – original draft:Equal; Writing – review & editing:Equal

SUPPLEMENTARY MATERIAL

A coupled ketoreductase-diaphorase assay for the detection of polyethylene terephthalate-hydrolyzing activity

María Gimeno-Pérez[a, b, c], James Finnigan[d], Coro Echeverria[e], Simon J. Charnock[d], Aurelio Hidalgo[a,b,c]* and Diana M. Mate[a, b,c]*

[a] Dr. M. Gimeno-Pérez, Dr. A. Hidalgo, Dr. D.M. Mate
Department of Molecular Biology
Universidad Autónoma de Madrid
Campus de Cantoblanco, Madrid 28049, Spain.
E-mail: ahidalgo@cbm.csic.es, diana.mate@uam.es

[b] Dr. M. Gimeno-Pérez, Prof. A. Hidalgo, Dr. D.M. Mate
Center of Molecular Biology "Severo Ochoa" (UAM-CSIC)
Nicolás Cabrera 1, Madrid 28049, Spain.
E-mail: ahidalgo@cbm.csic.es, diana.mate@uam.es

[c] Dr. M. Gimeno-Pérez, Prof. A. Hidalgo, Dr. D.M. Mate
Institute for Molecular Biology-IUBM
Universidad Autónoma de Madrid
Campus de Cantoblanco, Madrid 28049, Spain.
E-mail: ahidalgo@cbm.csic.es, diana.mate@uam.es

[d] Dr. J. Finnigan, Dr. S.J. Charnock
Prozomix Ltd.
Haltwhistle, Northumberland NE49 9HA, United Kingdom.

[e] Dr. C. Echeverria
Institute of Polymer Science and Technology
Spanish Research Council
Juan de la Cierva 3, 28006 Madrid, Spain.

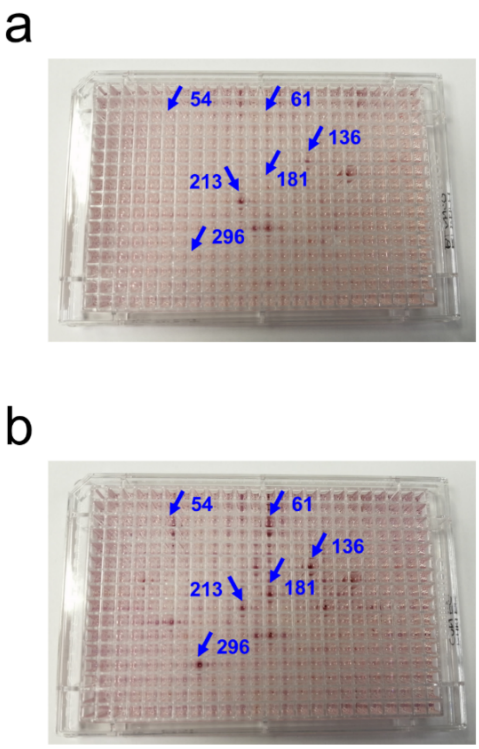

Figure S1. kREDY-to-go assay for identification of ketoreductases with ethylene glycol activity. (**a**) Control plate without substrate. (**b**) Reaction plate containing 0.5% (v/v) EG. Pictures were taken after 24 h of incubation at room temperature.

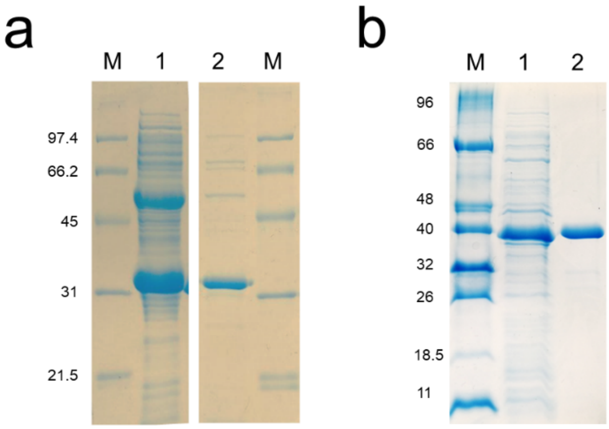

Figure S2. SDS-PAGE analysis of Cut190 S184P/R186S and KRED61. (**a**). SDS-PAGE analysis of Cut190 S184P/R186S. Lane 1: soluble fraction of *E. coli* cells expressing Cut190 S184P/R186S. Lane 2: Cut190 S184P/R186S purified by nickel-affinity chromatography. M: protein molecular ladder in kDa. (**b**). SDS-PAGE analysis of KRED61. Lane 1: soluble fraction of *E. coli* cells expressing KRED61. Lane 2: KRED61 purified nickel-affinity chromatography. M: protein molecular ladder in kDa.

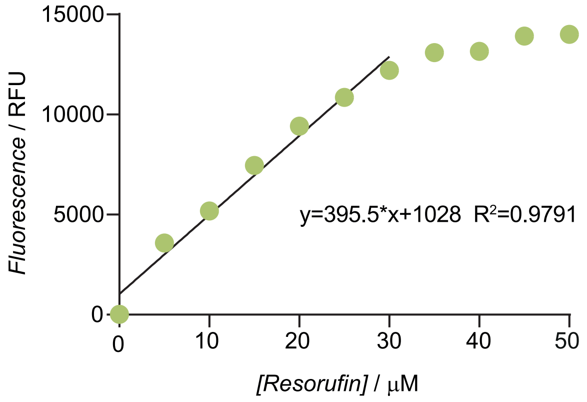

Figure S3. Calibration curve of resorufin in 25 mM Tris-HCl buffer pH 8.0.

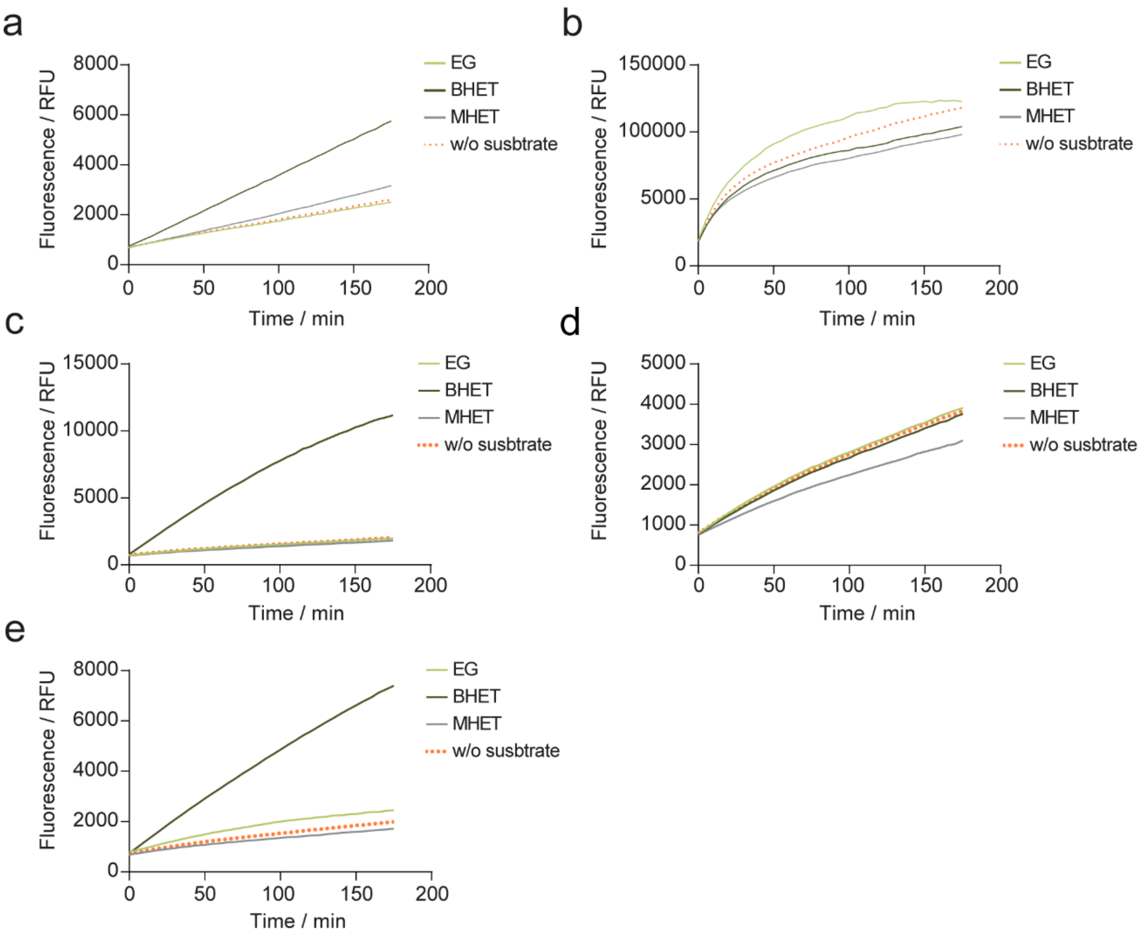

Figure S4. Time courses for the oxidation of 2.5 mM EG, MHET or BHET with EG-oxidizing KREDs in the coupled reaction with diaphorase. The activity was determined using lyophilized (1 mg/mL cell-free extract) KREDs #54 (**a**); #136 (**b**); #181(**c**); #213 (**d**) and #296 (**e**). The production of resorufin by the coupled reaction with diaphorase (0.1 U/mL) was monitored at room temperature in presence of KRED, $NADP^+$ (3 mM), diaphorase and resazurin (30 μM) in 25 mM Tris-HCl buffer pH 8.0. Controls without substrate were carried out in parallel.

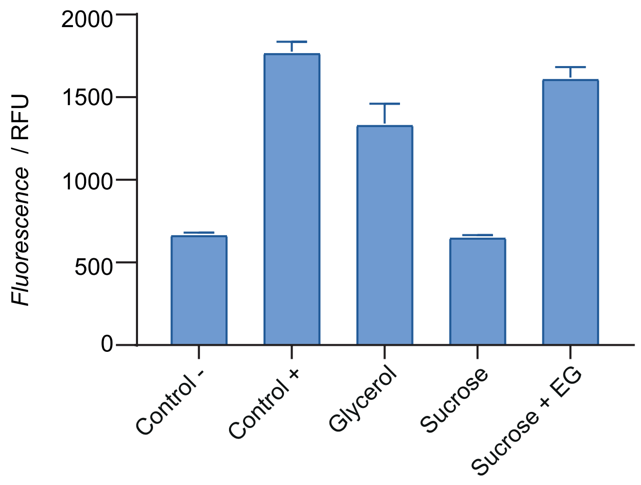

Figure S5. Effect of glycerol and sucrose in the coupled reaction KRED61-diaphorase. The stabilizers glycerol and sucrose were added at 0.1% and 100 mM to the coupled reaction and the signal fluorescent was evaluated at 90 min. Negative and positive controls were done without and with 45 mM EG, respectively.

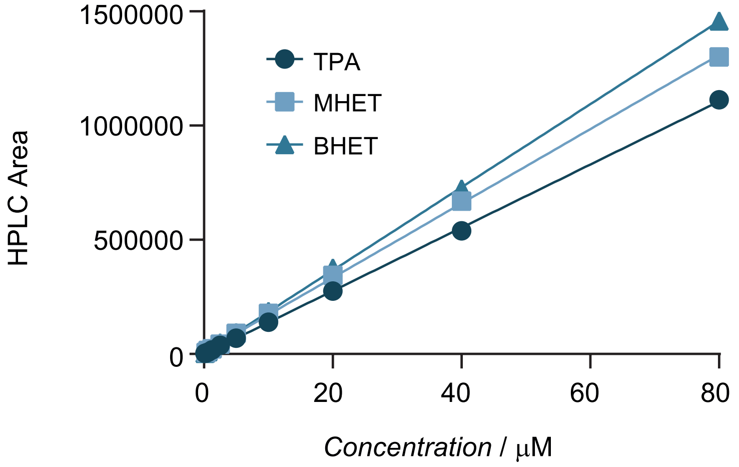

TPA: y=13828*x-285.8 R^2=0.9998

MHET: y=16265*x+8438 R^2=0.9997

BHET: y=18184*x+2400 R^2=0.9999

Figure S6. Calibration curves of TPA, MHET and BHET obtained by HPLC.

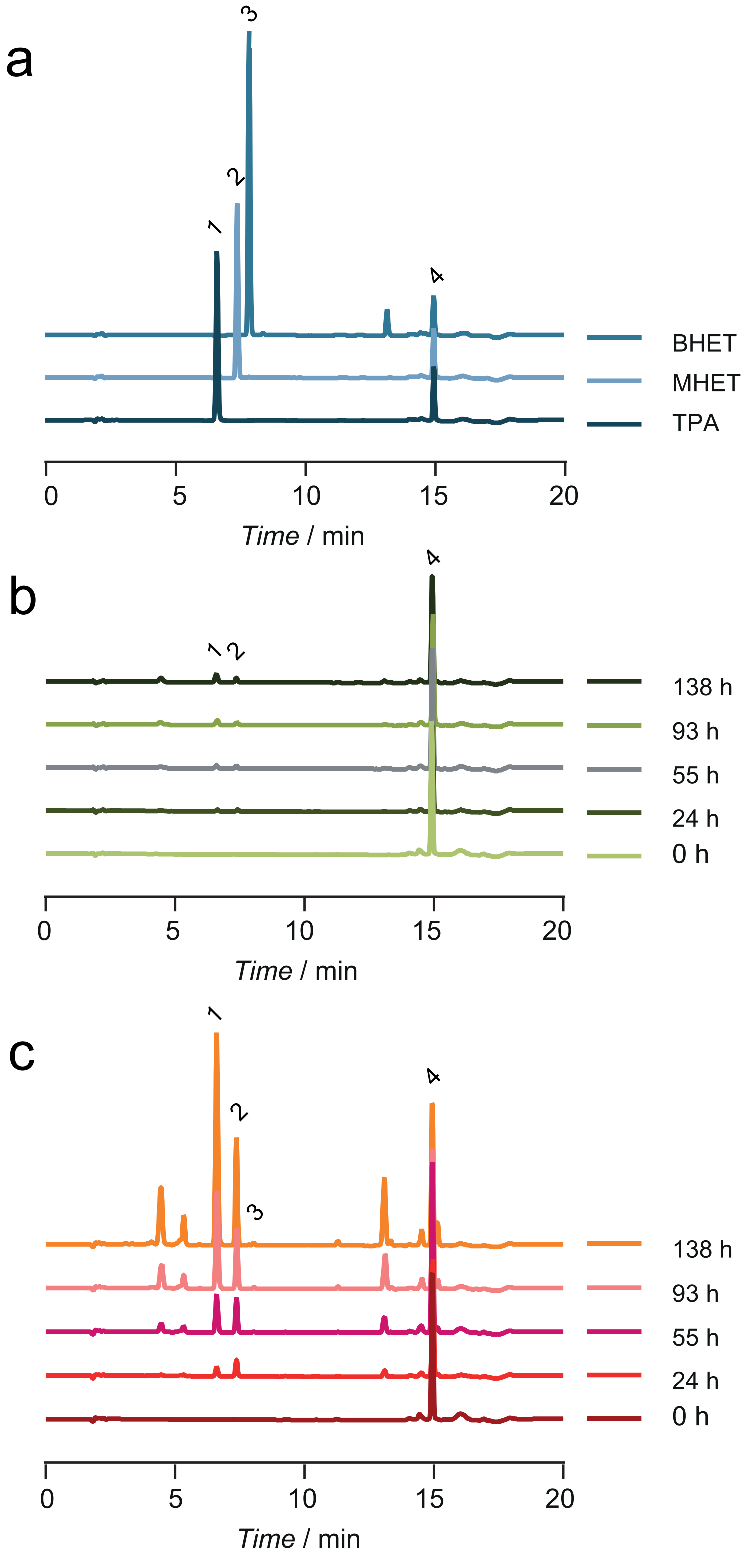

Figure S7. HPLC chromatograms. (**a**) Chromatograms of TPA, MHET, and BHET standards prepared for this analysis. (**b**) Chromatograms of the degradation reaction of PET films by Cut190 S184P/R186S without sucrose. (**c**) Chromatograms of the degradation reaction of PET films by Cut190 S184P/R186S with 1 M sucrose.

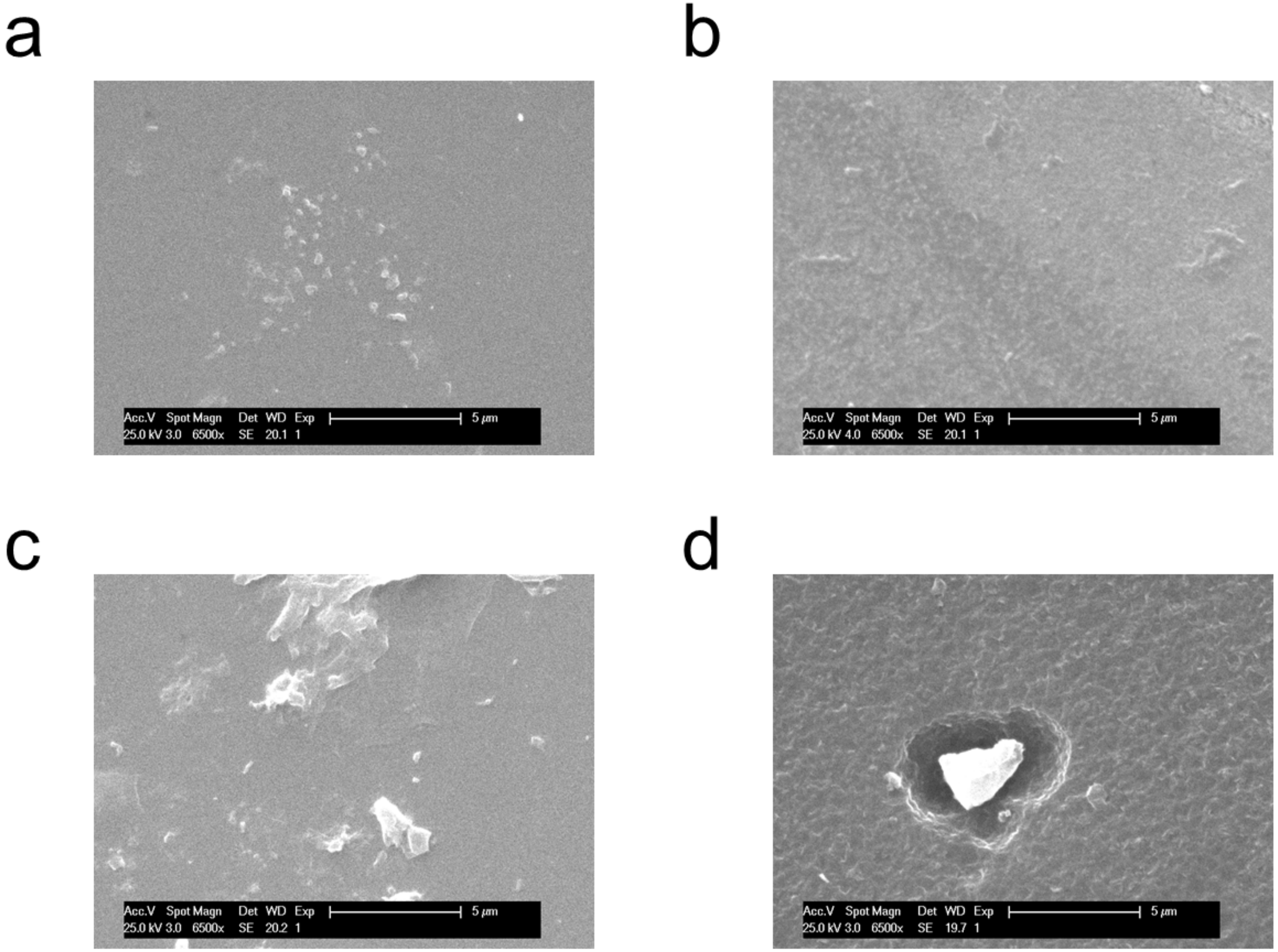

Figure S8. SEM images of PET films exposed to Cut190 S184P/R186S without (**b**) and with 1 M sucrose (**d**). **a** and **c** show negative controls without and with 1 M sucrose, respectively.

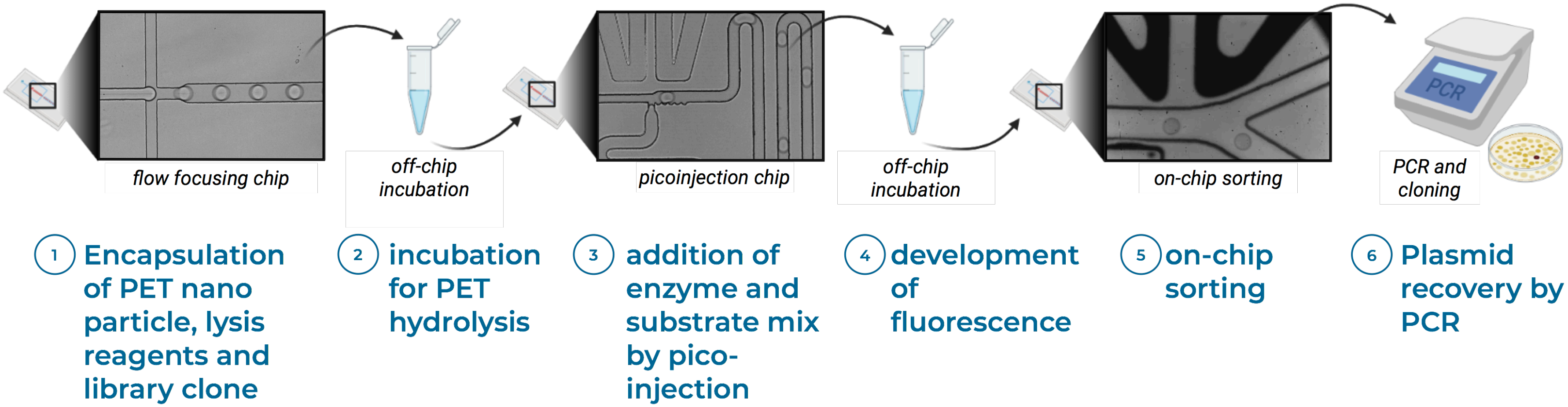

Figure S9. Proposed workflow for the implementation of the PET-hydrolase assay in droplets. A possible workflow for the determination of PET hydrolysing activity in droplets would comprise several on-chip and off-chip stages. First, a PET nanoparticle is encapsulated with the library clone expressing the candidate hydrolase in a water-in-oil (w/o) prior to cell lysis using typical lysis agents (BugBuster, lysozyme or the Lysis-on-Demand system). After sufficient incubation at 60 °C for PET hydrolysis to take place, the KRED+diaphorase reaction mix is added to the droplets by picoinjection or droplet fusion, following a 60-120 min incubation to reveal the presence of hydrolysis products before proceeding to on-chip droplet sorting and recovery of the DNA from the droplets by PCR or direct plating, according to the type of cell lysis used. Some modifications for added robustness of this proposed workflow may include the use of dodecyl resazurin or the use of a soluble formazan and an on-chip absorbance sorter (Pertinent references can be found in the main body of the article).
